# Supplementary material for: Strongly confined mid-infrared to terahertz phonon polaritons in ultrathin SrTiO3
Source: Sci Adv. 2025 Nov 19;11(47):eady7316. doi: 10.1126/sciadv.ady7316 (PMC12629183; doi:10.1126/sciadv.ady7316)
Supplement: Supplementary file 1 — Supplementary Text Figs. S1 to S7 [file sciadv.ady7316_sm.pdf]

Science Advances
AAAS

Supplementary Materials for

Strongly confined mid-infrared to terahertz phonon polaritons in ultrathin $SrTiO_3$

Peiyi He *et al.*

Corresponding author: Pu Yu, yupu@mail.tsinghua.edu.cn; Peng Gao, pgao@pku.edu.cn

Sci. Adv. **11**, eady7316 (2025)
DOI: 10.1126/sciadv.ady7316

This PDF file includes:

Supplementary Text
Figs. S1 to S7

Supplementary Text

1. Dispersion equation for small d

For an isotropic dielectric membrane of thickness d suspended in air, the symmetric and antisymmetric modes are governed by the following relations (*36, 53*)

$$\varepsilon k_0^z + k^z \tanh\left(\frac{-ik^z d}{2}\right) = 0 \text{ (symmetric)}$$
$$\varepsilon k_0^z + k^z \coth\left(\frac{-ik^z d}{2}\right) = 0 \text{ (antisymmetric)},$$
$$k_0^z = \sqrt{\left(\frac{\omega}{c}\right)^2 - \tilde{q}^2},\ k^z = \sqrt{\varepsilon\left(\frac{\omega}{c}\right)^2 - \tilde{q}^2}, \quad \text{(S1)}$$

where $\varepsilon = \varepsilon(\omega)$ is the complex dielectric function of the membrane, and $\tilde{q} = q(\omega) + i\kappa(\omega)$ is the complex wavevector.

For very small d, the modes of interest have large wavevector. Under large wavevector approximation, we have $k_0^z \approx k^z \approx \pm i\tilde{q}$. In the following equations, we have chosen the signs to ensure decaying solutions such that $\mathrm{Im}(k^z)$, $\mathrm{Im}(k_0^z)$ and $\mathrm{Im}(\tilde{q})$ are all positive, which were further verified after obtaining the solutions. The dispersion equations then simplify to:

$$\tanh\left(\frac{-\tilde{q}d}{2}\right) = -\varepsilon \text{ (symmetric)}$$
$$\coth\left(\frac{\tilde{q}d}{2}\right) = -\varepsilon \text{ (antisymmetric)}. \quad \text{(S2)}$$

Using the transformation relations of hyperbolic functions and taking into account the multivalued nature of the solutions, we obtain the following expressions for the complex wavevector $\tilde{q}$

$$\tilde{q} = \frac{2}{d}\operatorname{arctanh}\left(\frac{1}{\varepsilon}\right) - \frac{1}{d}(2m-1)\pi i \text{ (symmetric)}$$
$$\tilde{q} = -\frac{2}{d}\operatorname{arctanh}\left(\frac{1}{\varepsilon}\right) + \frac{1}{d}2n\pi i \text{ (antisymmetric)}. \quad \text{(S3)}$$

Considering modes that do not vanish rapidly, we have $m = 0$ for symmetric mode and $n = 0$ for antisymmetric mode:

$$\tilde{q} = \frac{2}{d}\operatorname{arctanh}\left(\frac{1}{\varepsilon}\right) + \frac{1}{d}\pi i \text{ (symmetric)}$$
$$\tilde{q} = -\frac{2}{d}\operatorname{arctanh}\left(\frac{1}{\varepsilon}\right) \text{ (antisymmetric)}. \quad \text{(S4)}$$

It is worth noting that in the small d limit, the real parts of the symmetric and antisymmetric modes share the same functional form (set to be positive for plotting), reproducing the expression given in the main text (Eq. 3): $q(\omega) = \mathrm{Re}[-\frac{2}{d}\operatorname{arctanh}\left(\frac{1}{\varepsilon}\right)]$.

We can also obtain the expression for the imaginary part of the wavevector as

$$\kappa(\omega) = \mathrm{Im}\left[\frac{2}{d}\operatorname{arctanh}(\varepsilon)\right] \text{ (symmetric)}$$
$$\kappa(\omega) = \mathrm{Im}\left[-\frac{2}{d}\operatorname{arctanh}\left(\frac{1}{\varepsilon}\right)\right] \text{ (antisymmetric)}. \quad \text{(S5)}$$

These results can also be derived from the Fabry-Perot quantization condition (*54*). Here we have derived the solution presented in the main text, which clearly demonstrates how the thickness d influences the PhP modes in the small-d limit. For comparison, Fig. S1 also shows the solutions

obtained by directly solving Eq. (S1) for different thicknesses, including the real part of wavevector $q(\omega)$, propagation length $L(\omega) = 1/(2\kappa(\omega))$, and factor $Q(\omega) = q(\omega)/\kappa(\omega)$. As the membrane thickness decreases, the Q factor first drops and then becomes nearly independent of thickness. This behavior can be understood from the fact that, in the small-d regime, both the real and imaginary parts of wavevector are squeezed by the $1/d$ scaling, so that their ratio no longer depends on d.

2. Mode mapping of localized PhP resonances

To further explore localized PhP resonances, we performed mode mapping on a freestanding $SrTiO_3$ membrane with a thickness of 30 nm. Two representative geometries were investigated: a protruding rectangular region with a side length of ~300 nm (Fig. S7A-C) and a triangular region with a side length of ~200 nm (Fig. S7D-F).

Since these protrusions are connected to the surrounding membrane, they do not constitute truly isolated blocks. Strictly speaking, the observed features are therefore not fully localized modes. Nevertheless, the energy-filtered EELS maps reveal distinct signatures that are consistent with our BEM simulations. For the rectangular geometry, the experimental maps (Fig. S7B) show that with increasing energy, the resonance hotspots gradually shift toward the corners, reminiscent of the dispersive nature of propagating PhPs (noting that the structure extends beyond the rectangular protrusion). For the triangular geometry, we observe a sequence of resonant features: at ~70 meV corresponding to a bulk-like mode, at ~75 meV showing strong localization at the corners, and at ~80 meV associated with edge resonances (Fig. S7E-F).

These results demonstrate that the high spatial resolution of STEM-EELS enables detailed mode mapping of PhPs, providing valuable insight into the behavior of confined resonances in nanostructured perovskite membranes. Such an approach is useful for understanding and optimizing the optical properties of nanostructured materials for advanced functionalities.

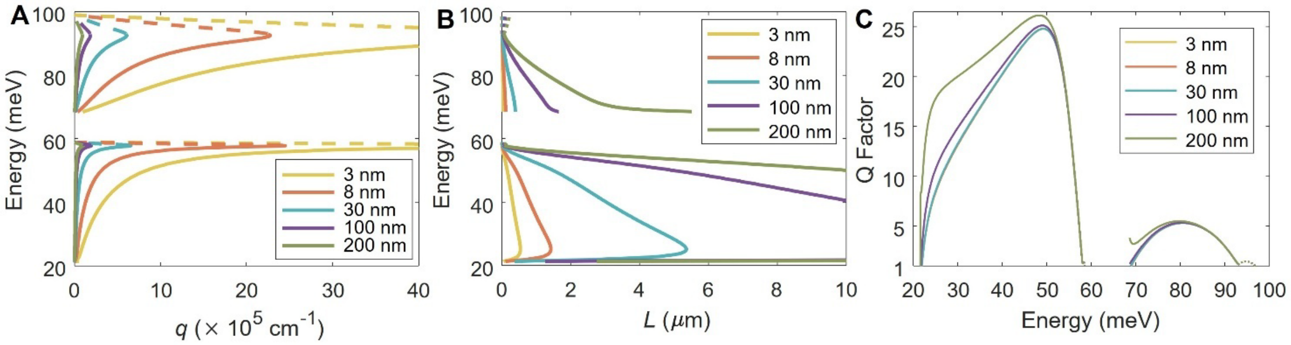

Fig. S1. Thin-film PhPs in $SrTiO_3$ membranes. Calculated (**A**) dispersion relations, (**B**) propagation lengths, and (**C**) quality factors of thin-film PhPs in freestanding $SrTiO_3$ membranes with different thicknesses. Dashed lines denote symmetric modes and solid lines denote antisymmetric modes.

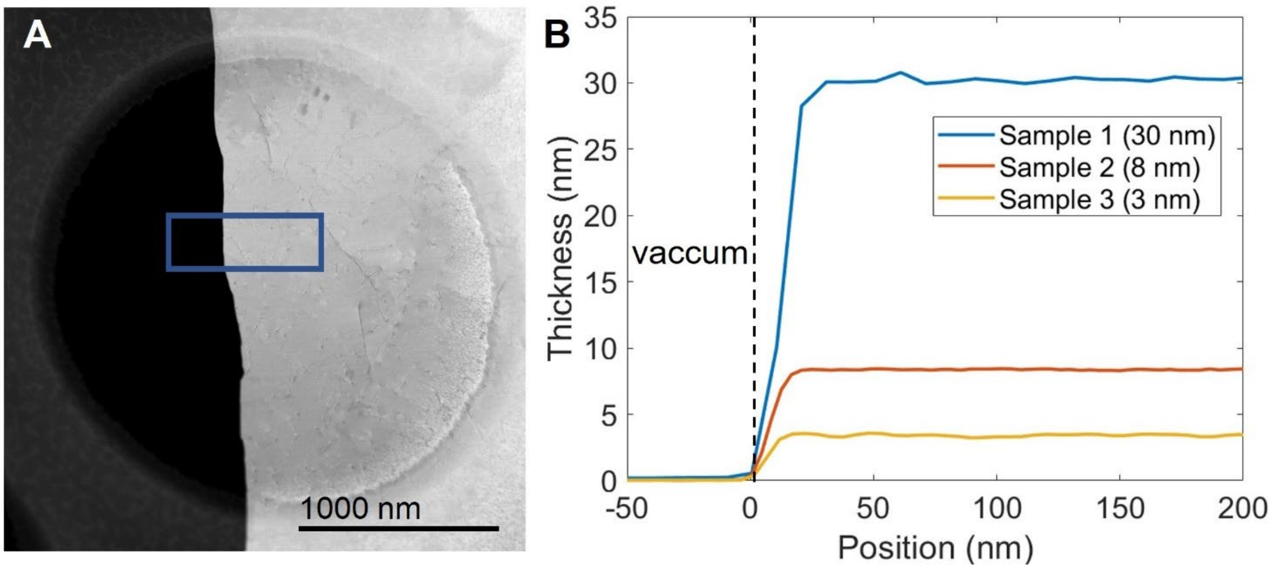

Fig. S2. Sample configuration. (**A**), HAADF image of a freestanding $SrTiO_3$ thin film, with part of the film resting on the carbon grid. The blue box indicates the STEM-EELS scanning region. (**B**), Thickness profiles of different samples determined by the log-ratio method, based on the ratio of plasmon losses to the ZLP intensity in EELS, obtained from scans spanning from vacuum to the sample interior.

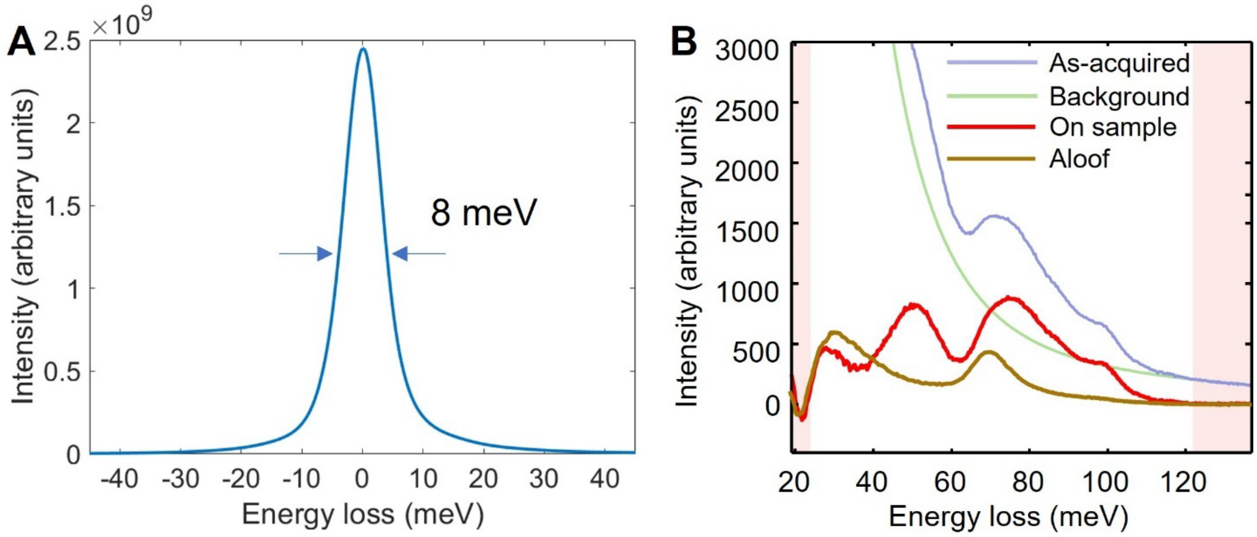

Fig. S3. Energy resolution and background subtraction in STEM-EELS measurements. (**A**), Measured zero-loss peak with a full width at half maximum of 8 meV, indicating the energy resolution of STEM-EELS measurements. (**B**), EELS spectra and the background subtraction. Purple: Raw data collected. Green: Fitted background using $\exp[P_3(x)]$ over two intervals. Red: Signal obtained after background subtraction. The above corresponds to the case where the electron beam passes through the sample. The brown curve represents the data obtained from the aloof configuration, processed in the same manner.

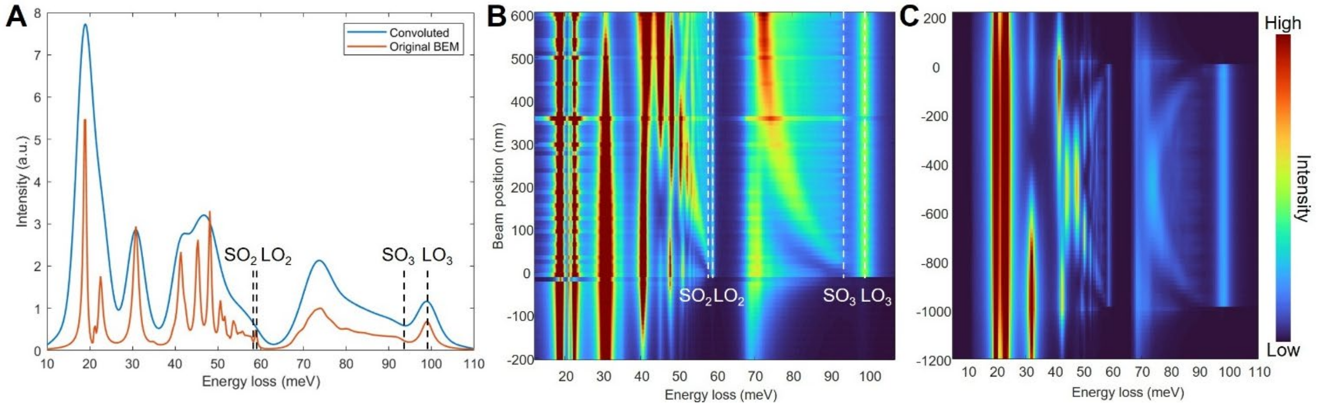

Fig. S4. BEM simulations. (**A**) Spectrum at 600 nm (from panel B) together with its convoluted counterpart. (**B**) Simulated EELS line scan corresponding to Fig. 2f before energy convolution. Modes satisfying $\boldsymbol{2qd + \pi/4 = 2n\pi}$ (with positive integers *n*) contribute to the feature in the figure. (**C**) Wide-range scan across a square plate of side length 1000 nm and thickness 30 nm. Due to the finite side length, access to lower-q, lower-energy modes is limited.

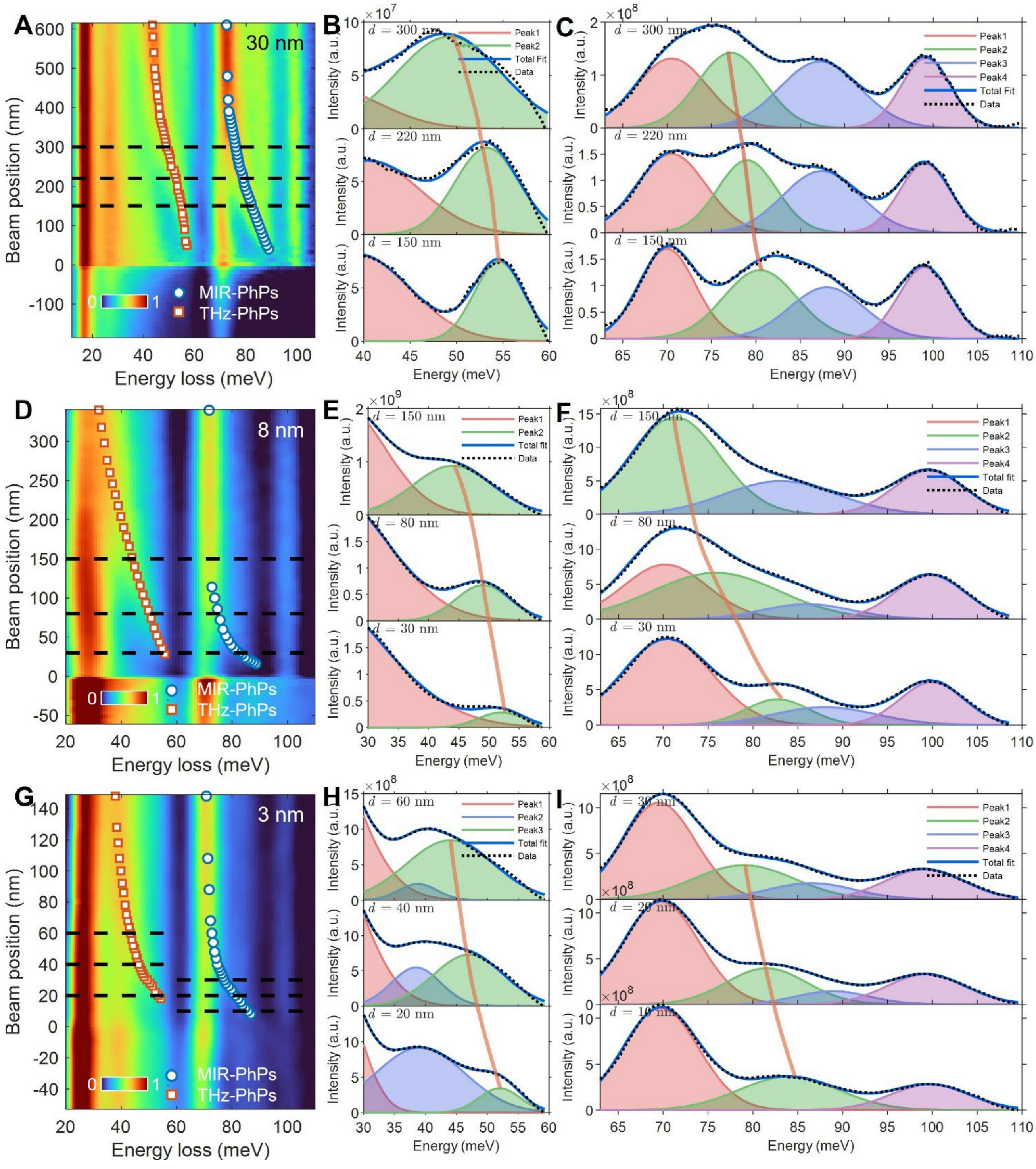

Fig. S5. Data extraction and fitting for PhPs in $SrTiO_3$ membranes. (**A** to **C**) 30 nm, (**D** to **F**) 8 nm, and (**G** to **I**) 3 nm membranes. (**A**, **D**, **G**) Line-scan EELS intensity maps as a function of beam position and energy loss. White circles denote MIR-PhP dispersions, and white squares denote THz-PhP dispersions, extracted from the subsequent fitting analysis. Note that for cases where the fits yielded the same energy, only the data point at the intermediate position was selected. Black dashed lines indicate the beam positions where representative spectra are analyzed. (**B**, **E**, **H**) Representative spectra in the THz range with multi-peak Gaussian fits. (**C**, **F**, **I**) Representative spectra in the MIR range with multi-peak Gaussian fits. Experimental data (black dotted lines) are decomposed into individual peaks (colored shaded areas), with the total fit shown as solid blue lines. The PhP-related peaks are highlighted

in green, and orange-red lines mark the shifts of their peak energies. The selected beam positions correspond to distances d from the boundary of (300, 220, 150) nm for 30 nm membranes, (150, 80, 30) nm for 8 nm membranes, and (60, 40, 20) / (30, 20, 10) nm for 3 nm membranes.

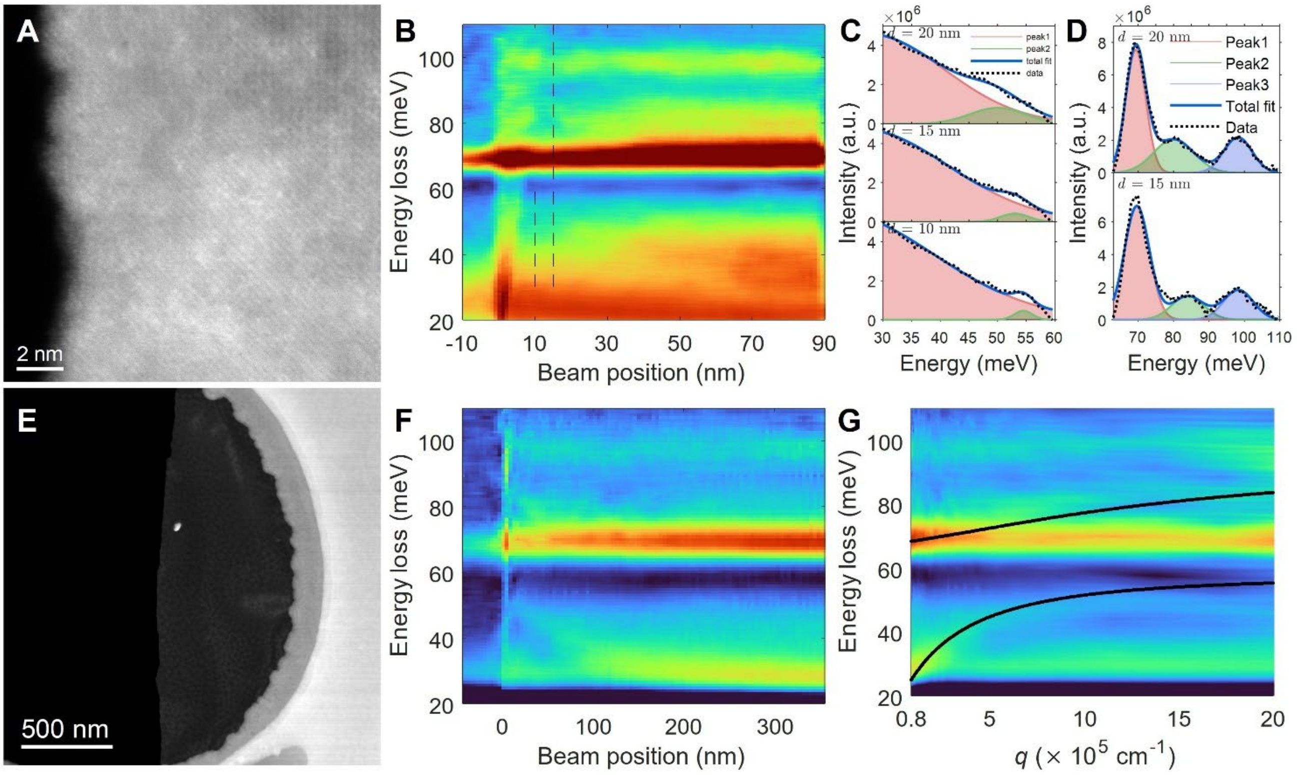

Fig. S6. Additional datasets for 3-nm $SrTiO_3$ membranes showing the effects of spatial sampling and field of view on PhP measurements. (**A**) STEM-HAADF image at the edge of the 3-nm membrane, revealing ~2-nm roughness. (**B**) EELS line-scan acquired with 1 nm per pixel across the edge. PhP features in the THz and MIR bands are visible. Black dashed lines mark the spectra plotted in (C to D). (**C**) Representative THz-band spectra with multi-peak Gaussian fits. (**D**) Representative MIR-band spectra with multi-peak Gaussian fits. (**E**) Overview STEM-HAADF image of the 3-nm membrane on a carbon grid. (**F**) Wide-range EELS line-scan (4 nm per pixel) over an extended field of view, enabling access to lower PhP energy. (**G**) PhP dispersion obtained from (F). Black curves are analytically calculated dispersion relations. These additional datasets were acquired with an updated direct electron detector and an optimized optical path, achieving an improved energy resolution of 5.6 meV. Edge roughness (~2 nm) limits the achievable confinement by introducing edge-position uncertainty (real-space to q mapping), perturbing modes and increasing high-q damping, and reducing fringe visibility/contrast. Random edge conditions make quantitative analysis more difficult. The reported confinement and group velocity values are conservative and represent reliably achievable levels.

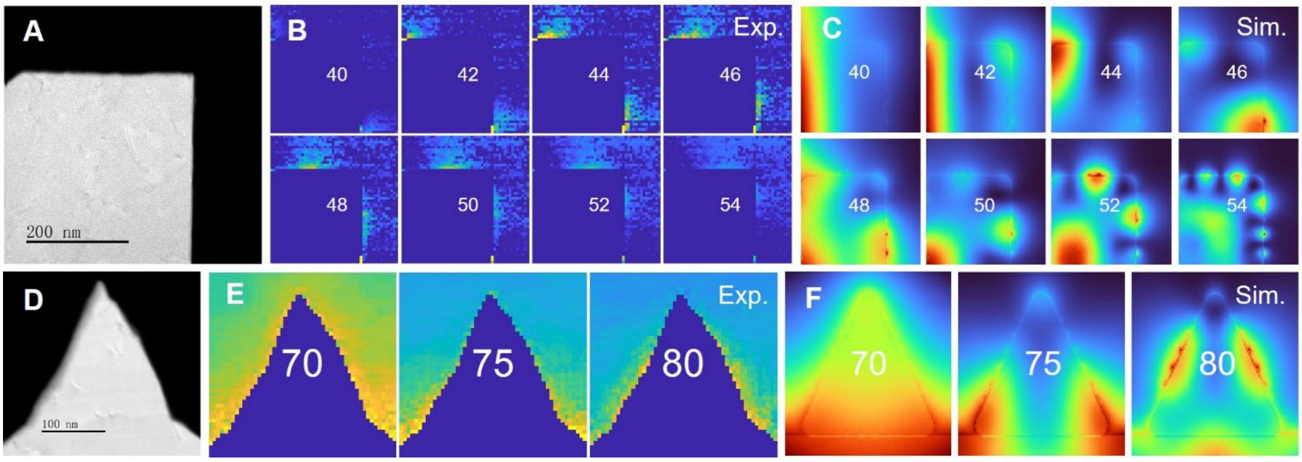

Fig. S7. Real-space mode mapping on a 30 nm thick $SrTiO_3$ film. (**A**), HAADF image of a protruding rectangle. (**B**), Energy-filtered EELS maps, with numbers representing the energy in meV (same for subsequent cases). (**C**), BEM simulation results corresponding to (B). (**D**), HAADF image of a protruding triangle. (**E**), Energy-filtered EELS maps. (**F**), BEM simulation results corresponding to (E).
